# Physicians’ preventive practices: more frequently performed for male patients and by female physicians

**DOI:** 10.1186/s12913-020-05136-2

**Published:** 2020-04-20

**Authors:** Raphaëlle Delpech, Géraldine Bloy, Henri Panjo, Hector Falcoff, Virginie Ringa, Laurent Rigal

**Affiliations:** 1 General Practice Department, Université Paris-Saclay, Le Kremlin Bicêtre, France; 2 LEDi, EA 7467, University of Burgundy Franche-Comté, Dijon, France; 3 CESP, INSERM, Paris-Saclay University, Paris-Sud University, UVSQ, Villejuif, France; 4 Institut National d’Études Démographiques (INED), Paris, France; 5 Société de Formation Thérapeutique du Généraliste, Paris, France

**Keywords:** Primary health care, Family practice, General practice, Gender, Preventive medicine, Preventive care, Patient-physician gender concordance, women’s health

## Abstract

**Background:**

We sought to analyze gender differences in General Practitioners’ (GP) preventive practices: variations according to the GP’s and the patient’s genders, separately and combined, and the homogeneity of GPs’ practices according to gender.

**Methods:**

Fifty-two general practitioners volunteered to participate in a cross-sectional study. A sample of 70 patients (stratified by gender) aged 40–70 years was randomly chosen from each GP’s patient panel. Information extracted from the medical files was used to describe the GPs’ preventive practices for each patient: measurements of weight, waist circumference, glucose, and cholesterol; inquiry and counseling about smoking, alcohol consumption, diet, and physical activity, and dates of cervical smears and mammographies. An aggregate preventive score was calculated to assess the percentage of these practices performed by each GP for patients overall and by gender. Mixed models were used to test for gender differences.

**Results:**

Questionnaires were collected in 2008–2009 for 71% of the 3640 patients and analyzed in June 2017.

Male patients and female GPs were associated with the most frequent performance of many types of preventive care. The aggregate preventive score was higher for male patients (OR = 1.60, 95% CI 1.47–1.75) and female GPs (OR = 1.35, 95% CI 1.05–1.73). There was no combined effect of the genders of the two protagonists. Female patients of male GPs appeared to receive preventive care least frequently and female GPs to deliver preventive care more consistently than their male colleagues.

**Conclusion:**

Physicians need to be aware of these differences, for both patient gender and their own.

## Background

Numerous studies have examined the influence of the gender of both the patient and the physician on the quality of preventive care dispensed. Some preventive procedures appear to be performed more frequently among men and others among women. Men thus appear to receive preventive cardiovascular care [1–3] and advice about tobacco and alcohol use [4] more frequently than women but diet and lifestyle advice less often [5, 6]. The physician’s gender also appears to be associated with the provision of preventive care. On the whole, women doctors appear to provide this care more often than their male colleagues, as demonstrated for cardiovascular prevention [1, 2], cancer screening [4, 7, 8], and vaccination [9, 10]. This result has nonetheless not been found systematically, and some aspects of prevention, such as prevention of overweight/obesity and advice about cessation or reduction of smoking and drinking [4], do not seem to differ according to the physician’s gender.

Beyond these somewhat contradictory observations, other aspects merit closer study. One of these is the combined effect of the gender of the patient and the doctor, currently the object of two hypotheses. First, effects of projection or identification might cause preventive care to be more frequent when the genders of each protagonist are concordant, as reported by a study of dietary-lifestyle advice and successful attainment of glycemic and blood-pressure goals [11]. Other studies, including some on this same dietary-lifestyle counseling topic [12, 13], have reported different results. Second, in the area of cardiovascular prevention, for example, practice differs less between patients of each gender when their primary care physicians are women compared with men [2]. To our knowledge, such analyses have not yet been conducted in other areas of prevention.

Finally, we assume that women physicians may have more consistent preventive practices among themselves than their male counterparts [14–16]. To our knowledge, the variation in physicians’ preventive practices according to their gender has never been studied.

The overall objective of this study was to analyze the gender differences in the preventive of French general practitioners (GPs). More specifically, we sought to analyze the association between their preventive practices, on the one hand, and, on the other, the patient’s gender, the GP’s gender, and the combined effect of both, as well as to study the homogeneity of GPs’ preventive practices according to their gender.

## Methods

### Design

This study is an ancillary analysis of data from an observational survey named *PrevQuanti,* designed to assess social inequalities in the preventive care — screening for breast and cervical cancer [17], tobacco, and alcohol consumption [18], and cardiovascular risk — provided by GPs to patients aged 40–74 years [2]. A power calculation determined that we would require 50 GPs and 70 patients per GP to be able to demonstrate social gradients for the types of preventive care studied [19]. PrevQuanti was conducted in 2008–09 among GPs who supervised students training in general practice during an internship at their office. We used email and telephone to recruit GPs working with two medical school departments of general practice in the Paris metropolitan area (who were paid 300 € for their time). For each participating GP, a random sample of 35 men and 35 women was drawn from their patient list (patients who had reported them to be their regular GP), furnished by the national health insurance fund. In practice, we used the “random” function of Excel for the random drawing of patients from the list for each GP. There were no exclusion criteria.

### GPs’ characteristics

We collected the following GP characteristics, through a self-administered questionnaire: age, sex, mean duration of consultations, mean number of consultations weekly, and office location.

### Patients’ characteristics

A data collection template was used to extract characteristics of these patients’ medical management from their files: number of visits during the past year, length of follow-up (management), and the preventive practices performed. A questionnaire mailed to patients’ homes (for self-administration) collected various social characteristics, such as educational level [20].

### Statistical analysis

In a preceding article we studied the assessment of cardiovascular risk for primary prevention according to the gender of patients and their GPs [2]. Here we continue our study of preventive care dispensed in general practice from a perspective focused on gender, but extend it on the one hand to other domains of prevention (overweight, alcohol consumption, diet, and gynecologic cancer screening) and on the other hand to the entire set of patients (without excluding those at high cardiovascular risk, as during our previous work). Binary dependent variables (that is, variables to be explained), obtained from the medical files, were used to describe the GPs’ preventive practices. These variables were selected from RAND’s Quality Assessment Tools, a set of quality indicators of preventive and chronic disease care developed and evaluated in the United States [21]. They are measures of process (more than result) and represent concrete activities that clinicians control rather directly [22]. Five domains of prevention (with 2 dependent variables per domain) were considered: weight management with weight and waist circumference measurements (ever documented in the file, regardless of when); substance use, with smoking and alcohol consumption status documented (ever documented in the file; smoking status was considered in our previous article); lifestyle recommendations, with provision of diet and physical activity advice (documented in the file within the past 3 years); cardiovascular risk with fasting blood glucose and cholesterol measurements (documented in the file within the past 5 years; both variables considered in the previous article); and gynecological cancer screening with cervical smear and mammography dates documented (ever documented in the file).We also constructed an aggregate preventive score. Calculated at the patient level, it was the percentage of the preventive practices performed among all the dependent variables applicable to both genders.

All of the analyses were performed with mixed logistic models with a random intercept, adjusted for characteristics of patients and GPs known to be associated with the dependent variables studied. The patient characteristics used for adjustment were: age (in 5-year groups), body mass index (BMI < 25 kg/m^2^ - [25–30] - > 30) [23, 24], educational level (did not pass the “bac” school-leaving exam, passed the “bac”, university level) [25], the annual number of visits (0, [1, 2], ≥3) [26, 27], the length of the patient-GP relationship ([0–1[, [1, 2], ≥3 years). The GP characteristics used for adjustment were: age (< 50 years,]50–60], > 60) [28], mean duration of consultation (≤ 20 min vs > 20), mean number of visits weekly ([50–70],]70–100], > 100) [26], and location of office (Paris vs suburbs) [26]. The absence of strong colinearity between these characteristics was verified by measuring Variance Inflation Factors (maximum: 2.3).

The dependent variables were analyzed first according to the gender of the patient and the GP (with comparison of the inter-GP variances among male and female GPs; our preceding article did not perform this analysis of variance) and then according to the gender of both combined into pairs.

The statistical analyses were performed with SAS software, v. 9.4. The advisory committee for information treatment for health research (commission nationale de l’informatique et des libertés) approved the study, and all patients signed informed consents.

## Results

### Description of GPs

The first 52 GPs who volunteered to participate were included in the study. We review here only the essential aspects of the description of the GPs [19] and their comparison according to gender [2] which have already been presented. Their mean age was 55 years (standard deviation (SD) = 6), and 63% of them were men. Their mean duration of consultations was 21 min (SD = 4.8), and on average they saw 92 (SD = 23) patients weekly. Moreover, the consultations by women GPs lasted longer than those of their male colleagues (*P* = 0.02).

### Description of patients

For the 3640 randomly selected patients, GPs returned 3600 questionnaires (98.8%), and patients 2605 (71.5%). Finally, data were collected from both the patient and GP for 71.4% (*n* = 2599) of the patients included. Patients’ mean age was 53.9 years (SD = 9.5) (Table 1). Men had been seeing their GPs for a significantly shorter period than the women and had a significantly higher body mass index (BMI).

**Table 1:** Characteristics of male and female patients (*N* = 2599)

		Male patients (*N* = 1259) *n* (%)	Female patients (*N* = 1340) *n* (%)	*P*
GP gender	Male	782 (62.1)	844 (63.0)	0.65
	Female	477 (37.9)	496 (37.0)	
Age (years)	[40–50[	453 (36.0)	499 (37.2)	0.28
	[50–60[	390 (31.0)	425 (31.7)	
	[60–75[	416 (33.0)	416 (31.0)	
Duration of GP-patient relationship (years)	[0–1]	91 (7.3)	77 (5.81)	**0.007**
	]1–3]	439 (35.2)	407 (30.7)	
	> 3	717 (57.5)	841 (63.5)	
Number of consultations annually	0	186 (14.8)	169 (12.7)	0.09
	1	170 (13.5)	177 (13.3)	
	2	209 (16.6)	193 (14.5)	
	> 2	691 (55.0)	794 (59. 6)	
BMI^a^ (kg/m^2^)	≤ 25	571 (46.6)	846 (64.9)	**< 0.001**
	]25–30]	502 (41.0)	288 (22.1)	
	> 30	153 (12.5)	170 (13.0)	
Educational level	University year 1–2	164 (13.3)	190 (14.5)	0.46
	Passed ‘bac’^b^	384 (31.1)	424 (37.3)	
	University year 3–4	687 (55.6)	700 (53.3)	

^a^ Body mass index

^b^ ‘bac’ is the baccalaureate examination for leaving secondary school

### Gender differences

Overall, male patients received preventive care significantly more often than women (Table 2). These differences were most marked in the domain of substance use. Moreover, preventive practices were more frequent among women GPs than their male colleagues. These gender differences were nonetheless significant only for smoking status, cardiovascular risk variables, and gynecological cancer screening. The aggregate preventive score was higher for male patients (odds ratio (OR) 1.60, 95% confidence interval (95% CI) 1.47–1.75, *P* <  10^− 4^) and female GPs (OR 1.35, 95% CI 1.05–1.73, *P* = .02).

**Table 2:** GPs’ prevention practices according to the gender of patient and GP (*N* = 2599)

	Gender differences: Patients				Gender differences: GPs				Inter-GP variances		
	Male patients (*N* = 1259) *n* (%)	Female patients (*N* = 1340) *n* (%)	OR [95% CI]^a^ (ref: female)	*P*	Male GPs (*N* = 1626) *n* (%)	Female GPs (*N* = 973) *n* (%)	OR [95% CI]^a^ (ref: male)	*P*	Variance between male GPs	Variance between female GPs	*P*
**Weight management**											
Weight measurement	876 (69.6)	891 (66.5)	1.26 [1.10–1.45]	**0.001**	1097 (67.5)	670 (68.9)	1.12 [0.47–2.63]	0.78	1.12	2.00	0.11
Waist circumference measurement	540 (42.9)	510 (38.1)	1.34 [1.08–1.66]	**0.008**	704 (43.3)	346 (35.6)	1.17 [0.43–3.18]	0.75	1.99	2.14	0.90
**Substance use**											
Smoking status documented	733 (58.2)	610 (45.5)	1.99 [1,65-2,40]	**< 10**^**−4**^	781 (48.0)	562 (57.8)	2.06 [1.11–3.84]	**0.02**	1.13	0.53	0.33
Alcohol use status documented	397 (31.5)	243 (18.1)	2.68 [2,15-3,35]	**< 10**^**−4**^	355 (21.8)	285 (29.3)	1.71 [0.76–3.86]	0.21	0.63	1.97	0.055
**Lifestyle recommendations**											
Received diet advice^b^	480 (38.1)	377 (28.1)	1.67 [1.37–2.03]	**< 10**^**−4**^	358 (36.8)	499 (30.7)	1.15 [0.70–1.91]	0.58	0.87	0.22	**0.03**
Received physical activity advice^b^	323 (24.1)	434 (34.5)	1.80 [1,46–2.22]	**< 10**^**−4**^	310 (31.9)	447 (27.5)	1.13 [0.56–2.30]	0.72	1.90	0.47	**0.015**
**Cardiovascular risk**											
Fasting blood glucose measurement^c^	858 (68.2)	848 (63.3)	1.47 [1.20–1.80]	**2.10**^**−4**^	1045 (64.3)	661 (67.9)	1.61 [1.05–2.45]	**0.03**	0.83	0.00	**10**^**−4**^
Cholesterol measurement^c^	774 (61.5)	705 (52.6)	1.66 [1.36–2.01]	**< 10**^**−4**^	925 (56.9)	554 (56.9)	1.52 [1.01–2.28]	**0.045**	0.71	0.002	**0.002**
**Gynecological cancer screening**											
Cervical smear date documented	–	–	–	–	169 (10.4)	167 (17.2)	2.94 [1.44–5.93]	**0.004**	1.45	0.38	0.07
Mammography date documented	–	–	–	–	300 (18.5)	221 (22.7)	2.14 [1.25–3.69]	**0.007**	0.47	0.35	0.75

a Odds ratios adjusted for patient variables (age, body mass index, number of consultations annually, duration of GP-patient relationship in years, and educational level) and for GP variables (age, office location, mean duration of consultations in minutes, and mean number of consultations weekly), followed by their 95% confidence intervals

^b^ within the previous three years

^c^ within the previous five years

Reading the table: each line corresponds to the results of the analysis of the variable of interest in the first column. Columns 2 to 5 (patients’ gender differences) present the differences according to patient gender (columns 2 and 3 combine all GP genders); Columns 6 to 9 (GPs’ gender differences) present the differences according to GP gender (columns 6 and 7 combine all patient genders); and finally, the last 3 columns (inter-GP variance), present the differences in inter-physician variance between men and women GPs

Except for weight management, the preventive practices explored varied significantly according to the gender composition of the patient-GP pair (Table 3). We thus observed a gradient according to the gender composition of these pairs: the woman patient-male GP pairs systematically had the least frequent preventive practices; the next most frequent was the matching female pairs (patient and GP both women), and then the male pairs. Finally, the male patient-female GP pairs had the most frequent prevention practices.

**Table 3:** GPs’ prevention practices and aggregate preventive score according to the gender composition of the patient-GP pair (*N* = 2599)

	Gender composition of the patient-GP pair^d^	%	OR [95% CI]^a^	*P*
Weight measurement	F/M	66.0	1	0.11
	F/F	67.3	1.76 [0.60–5.20]	
	M/M	69.1	1.25 [0.95–1.66]	
	M/F	70.4	1.76 [0.60–5.21]	
Waist circumference measurement	F/M	33.3	1	**0.045**
	F/F	32.3	1.07 [0.40–2.92]	
	M/M	45.3	1.25 [0.95–1.64]	
	M/F	39.0	1.60 [0.60–4.34]	
Smoking status documented	F/M	41.8	1	**< 10**^**−4**^
	F/F	51.8	**2.18 [1.16–4.08]**	
	M/M	54.7	**2.07 [1.64–2.62]**	
	M/F	63.9	**4.04 [2.14–7.63]**	
Alcohol use status documented	F/M	15.6	1	**< 10**^**−4**^
	F/F	17.3	1.76 [0.76–4.05]	
	M/M	28.5	**2.73 [2.05–3.64]**	
	M/F	36.5	**4.59 [2.01–10.47]**	
Received diet advice^b^	F/M	26.3	1	**< 10**^**−4**^
	F/F	31.3	1.03 [0.61–1.76]	
	M/M	35.4	**1.54 [1.19–1.98]**	
	M/F	42.6	**1.95 [1.15–3.31]**	
Received physical activity advice^b^	F/M	22.3	1	**< 10**^**−4**^
	F/F	27.2	1.82 [0.57–2.44]	
	M/M	33.1	**1.86 [1.42–2.44]**	
	M/F	36.7	2.04 [0.99–4.18]	
Fasting blood glucose measurement^c^	F/M	61.7	1	**2.10**^**−4**^
	F/F	65.9	1.51 [0.96–2.36]	
	M/M	67.0	**1.40 [1.08–1.80]**	
	M/F	70.0	**2.41 [1.52–3.84]**	
Cholesterol measurement^c^	F/M	53.4	1	**10**^**−4**^
	F/F	51.2	1.24 [0.80–1.91]	
	M/M	60.6	**1.40 [1.09–1.78]**	
	M/F	62.9	**2.69 [1.72–4.20]**	
Preventive aggregate score	F/M	41.1	1	**< 10**^**−4**^
	F/F	43.7	1.27 [0.95–1.69]	
	M/M	49.2	**1.42 [1.31–1.53]**	
	M/F	52.8	**1.99 [1.48–2.66]**	

^a^ Odds ratios adjusted for patient variables (age, body mass index, number of consultations annually, duration of GP-patient relationship, and educational level) and for GP variables (age, office location, mean duration of consultations, and mean number of consultations weekly), followed by their 95% confidence intervals

^b^ within the previous three years

^c^ within the previous five years

^d^ numbers in pair sets: F/M = 496; F/F = 782; M/M = 477; M/F = 844

Regardless of the preventive practice considered, the amplitude of the differences between the patients of each gender was globally similar for male and female GPs. For the aggregate preventive score, the OR for male compared with female patients of male GPs was 1.42 and of female doctors, 1.57 (= 1.99/1.27). These results were confirmed by non-significant findings for the tests of interaction between the patients’ and GPs’ genders (results not shown).

### Inter-GP variance

In the models adjusted for patient characteristics (i.e., taking into account a possible effect of the composition of the patient panels for these characteristics) and GP characteristics, the variance between male GPs was greater than that between women GPs for 7 of the 10 preventive practices analyzed and significantly greater for 4 of them (Table 3). The preventive practices of female GPs were more consistent and homogeneous than those of their male colleagues in terms of lifestyle recommendations and in the cardiovascular risk domain.

## Discussion

Our results show that male patients and female GPs are associated with the most frequent performance of numerous types of preventive care. There was no combined effect of the two protagonists: the effect of the patient’s gender is identical among male and female GPs; similarly, the effect of the GP’s gender is identical among male and female patients. Accordingly, women patients of male GPs appear to be the patient group least frequently receiving the types of preventive care we studied. Furthermore, it does not appear that the concordance of patient and GP gender influences preventive practices. Finally, women GPs seem to be more consistent in their delivery of preventive practices than their male colleagues.

The results according to patient gender reinforce the findings of studies of the inequality of preventive care that disadvantages women. We must nonetheless point out that our result for the aggregate preventive score disagrees with that of a study conducted among a younger sample of Americans (mean age 45 years); this disparity may be related to interaction between patient age and gender for prevention or to differences in the organization of care between the two countries [29].

Two complementary hypotheses may explain our observations favorable to male patients. The first posits that they may receive more preventive care because epidemiologic evidence indicates that they need it most [30, 31]. According to the second hypothesis, GPs target men for prevention more often because they consider them to be less informed, less aware of risks, and less receptive. It is difficult to determine which of these hypotheses is predominant. The two together probably persuade GPs that the stakes of preventive care are higher for men.

Although women’s rates of smoking and drinking [32] are tending to catch up with those of men (the consumption of these two substances increased among women and remained stationary among men, especially for smoking) [33–35], it is in the area of substance use that physicians’ preventive practices differ most between genders [36]. This may correspond to their lack of knowledge about recent modifications in these behaviors or to the application of a double standard, that is, a situation in which identical behaviors are judged differently as a function of gender. Excessive alcohol consumption, for example, is much more stigmatizing, even taboo, among women [37]. More or less consciously, such social representations may dissuade physicians from questioning their women patients about their alcohol consumption [38].

Our results about the GP’s gender are consistent with the literature [1–4] and underline the positive influence of women physicians in the area of preventive care. While they provide more of this type of care than their male counterparts, only half of these differences are statistically significant. This lack of significance may be due to the relatively small number of GPs, which limits the power of statistical tests (in hierarchical models with random effects, power is linked above all else to the number of GPs analyzed) [39, 40]. For the same reason, the greater homogeneity of preventive practices among women GPs, measured by the variance between them, should perhaps be analyzed in a larger GP sample.

Several sociological explanations help to interpret our findings related to GP gender. Educational practices in school promote originality more in boys and obedience in girls [41, 42] and may thus contribute to the propensity of male GPs to distance themselves from guidelines and that of women to comply with them. The traditional social roles of women also tend to construct a relation to time more oriented toward the planning of tasks and marked by a concern about consequences, which tends to be an asset in the area of prevention. Finally, although women today comprise a substantial portion of the medical world, their entry into this professional universe dominated by men is nonetheless recent [43]. In a quest for legitimacy, they may have had to demonstrate their willingness to conform to professional rules and especially guidelines [44]. Next, specifically for gynecological cancer screening, it is possible that the lack of investment by male GPs may be in part due to their female patients’ preference to have these tests performed by women [45, 46]. Finally, another part of these gender-based differences in preventive practices may be due to women’s more thorough recording of their practices. Women’s dispositions related to writing (effective note-taking acquired during their student life [44], writing lists and managing family organization in the domestic sphere) and differences in their organization of their professional practice (shorter working hours leading to greater file-sharing) [47] may combine to makes preventive management by women more traceable.

Our simultaneous analysis of patient and GP gender does not show that their concordance influenced care. Our observations thus do not support the hypothesis that a mirror-effect between patient and GP favors quality care. Moreover, although women GPs appear to provide more preventive care, they do so in a manner as inegalitarian, that is, favoring male patients, as their male colleagues. These differences raise issues beyond their ethical aspects, for recent work has demonstrated that some risk factors have a stronger, more negative effect on women than men. This is the case, for example, for diabetes as a cardiovascular risk factor [48] and for smoking and lung cancer [49].

Our study has also some strengths: its setting in general practice, different from the preceding studies, which have taken place essentially in hospitals or with specialists; consideration of the hierarchical structure and the non-independence of patients with mixed models; and the numerous adjustments for patient and GP characteristics known to be associated with prevention, which limit the possibility of residual confounding.

Our study also has several limitations. First, our analyses are not adjusted for patients’ individual history of diabetes and cardiovascular disease, although these are factors that underlie physicians’ preventive practices. We conducted sensitivity analyses by adjusting for this history and determined that it did not modify our results. Next, it did not take into account the health behaviors of the GPs, which are nonetheless likely to modify their preventive practices. Male GPs smoked more than their female colleagues [50, 51] and the current or previous smoking status of doctors affects their investment in smoking cessation [51]. Accordingly, not considering this type of characteristic is likely to lead to overestimating the differences between male and female GPs.

In France, prevention is one of the missions explicitly assigned to GPs. There is no type of facility or practice specifically devoted to prevention, and the use of practice nurses in GPs’ offices is just barely beginning in this country. Although access to specialists is relatively easy, only gynecologists will contribute to the gynecological cancer screening of a significant proportion of the population. Although these specificities related to health care in France may present problems about the generalization of our results to other countries, the fact that French GPs are relatively alone in ensuring preventive care enables the study of the influence of their gender about the preventive care dispensed. Moreover, the epidemiologic data about healthy behaviors globally less adopted by men and the male domination of the medical world, that is, the low concentration of women physicians within some specializations [52, 53] and their limited access to leadership positions in academic medicine [54, 55] are widely shared in much of the West. Consequently, although the male-female gaps observed may vary from one country to another, our results are probably at least qualitatively generalizable.

In this work, we adopted an analysis founded on equality (each managed the same) because the guidelines do not indicate any reason for different management according to the patients’ gender. Equity (to each according to his/her need) might also be an interesting point of view for this analysis, insofar as the needs for prevention are not necessarily the same for men and women. This may lead us to subsequent work on this topic.

## Conclusion

In conclusion, physicians, especially those providing primary care, must be made aware of these differences in their preventive practices, in relation to both their patients’ gender and their own. They must realize that the way they approach prevention, health behaviors, and patients’ risky practices is rarely neutral in relation to gender and that it directly affects their medical activities. Although the increasing feminization of medicine should result in the increased use of preventive care, the differences in practices prejudicial to women may well continue, independently of demographic changes. To improve this situation, the influence of sex and gender (in their biological and social dimensions) on health and illness and its implications for practice should be covered more thoroughly in the medical school curriculum.

## Data Availability

The datasets used and/or analysed during the current study are available from the corresponding author on reasonable request. The English questionnaires are available upon request.

## Abbreviations

BMI: Body mass index
GP: General practitioner
